# Population-Based Study of Smoking Behaviour throughout Pregnancy and Adverse Perinatal Outcomes

**DOI:** 10.3390/ijerph10093855

**Published:** 2013-08-27

**Authors:** Deirdre J. Murphy, Clare Dunney, Aoife Mullally, Nita Adnan, Richard Deane

**Affiliations:** 1 Academic Department of Obstetrics & Gynaecology, Coombe Women and Infants University Hospital & Trinity College Dublin, Dublin 8, Ireland; E-Mails: cdunney@coombe.ie (C.D.); amullally@coombe.ie (A.M.); richarddeane@physicians.ie (R.D.); 2 Coombe Women & Infants University Hospital, Dublin 8, Ireland; E-Mail: nita_adnan@hotmail.com

**Keywords:** smoking cessation, prospective cohort study, pregnancy, perinatal outcomes

## Abstract

There has been limited research addressing whether behavioural change in relation to smoking is maintained throughout pregnancy and the effect on perinatal outcomes. A cohort study addressed lifestyle behaviours of 907 women who booked for antenatal care and delivered in a large urban teaching hospital in 2010–2011. Adverse perinatal outcomes were compared for “non-smokers”, “ex-smokers” and “current smokers”. Of the 907 women, 270 (30%) reported smoking in the six months prior to pregnancy, and of those 160 (59%) had stopped smoking and 110 (41%) continued to smoke at the time of the first antenatal visit. There was virtually no change in smoking behaviour between the first antenatal visit and the third trimester of pregnancy. Factors associated with continuing to smoke included unplanned pregnancy (OR 1.9; 95% CI 1.3, 2.9), alcohol use (OR 3.4; 95% CI 2.1, 6.0) and previous illicit drug use (OR 3.6; 95% CI 2.1, 6.0). Ex-smokers had similar perinatal outcomes to non-smokers. Current smoking was associated with an average reduction in birth weight of 191g (95% CI −294, −88) and an increased incidence of intrauterine growth restriction (24% *versus* 13%, adjusted OR 1.39 (95% CI 1.06, 1.84). Public Health campaigns emphasise the health benefits of quitting smoking in pregnancy. The greatest success appears to be pre-pregnancy and during the first trimester where women are largely self-motivated to quit.

## 1. Introduction

Smoking is one of the most important modifiable causes of poor pregnancy outcomes, and is associated with increased risks of maternal, fetal and infant morbidity and mortality across populations [1,2,3,4]. Smoking in pregnancy is strongly associated with poverty, low educational attainment, poor social support and psychological stress, factors that are in themselves associated with adverse perinatal outcomes [3,5,6,7]. A global report on preterm birth and stillbirth reported evidence for only two interventions that prevent preterm birth, one of which was smoking cessation [2]. Many women continue to smoke in pregnancy, despite recommendations that they should quit in order to minimise potential risks to the fetus and newborn infant [8]. Intervention-based studies have demonstrated success in reducing maternal smoking later in pregnancy but limited success in relapse prevention [5]. Few population-based studies have addressed smoking behaviour prospectively from pre-pregnancy up until delivery outcome.

The aim of this study was to use a cohort of women booking for antenatal care and delivering in a Dublin maternity hospital to investigate the behavioural changes reported in relation to smoking pre-pregnancy, at the time of the first antenatal visit and in the third trimester of pregnancy and whether this affects adverse perinatal outcomes. This study was part of a larger study addressing “Lifestyle Behaviours in Pregnancy” including smoking, alcohol, illicit drug use, diet and exercise. The main hypothesis was to establish whether women who quit smoking in early pregnancy remain ex-smokers throughout pregnancy and whether this results in improved perinatal outcomes.

## 2. Methods

### 2.1. Sample

A prospective cohort study was carried out including women who booked for antenatal care and delivered in a large Dublin maternity hospital between November 2010 and December 2011. The maternity hospital booked over 9,500 women for maternity care in 2010. Women were eligible to be included if they had a singleton pregnancy, were aged 18 years or above and understood English. The aim was to invite every eligible woman to participate in the study, however given resource limitations and the range of settings for booking visits, a pragmatic approach was used by research staff to recruit from settings that had the greatest numbers of women booking on a given day. An initial sample size of 1,000 participants was planned, based on analyses from a previous study of alcohol exposure in pregnancy [9]. Data were collected in two phases; firstly at the booking visit by a structured interview and secondly, during the third trimester of pregnancy by a self-administered postal questionnaire. The sample size was inflated to 1,300 when a lower response rate to the third trimester questionnaire became apparent.

### 2.2. Recruitment

A list of women booking each day was obtained by members of the research team. Information leaflets were distributed to all eligible women as they waited for their booking visit in the antenatal clinic. After considering the study information, women interested in participating made contact with a member of the study team and were taken to a quiet area in the antenatal clinic. Consent to participate was discussed and written consent was given. To facilitate completion of the recruitment interview the woman and researcher remained in the quiet area and partners or accompanying friends were asked to remain in the main waiting area. This was to encourage women to be honest when answering questions of a sensitive nature. The women were asked the questions in a predetermined order and the interviews took between 5 to 10 min on average. When the interview was complete women were thanked for their participation and reminded that they would be receiving a third trimester postal questionnaire when they reached 28–32 weeks of pregnancy. The postal address of participants was confirmed at this stage. To protect confidentiality questionnaires were anonymised by allocating each participant a unique study number. To facilitate follow-up corresponding names were stored separately in a locked office with access only by members of the research team. Recruitment continued until the sample size of 1,300 women was achieved. 

### 2.3. Data Collection

Detailed information was gathered on lifestyle behaviours including smoking, alcohol intake, diet, infant feeding intention, and exercise. The interview schedule was developed by the multidisciplinary team and used validated questionnaires where possible such as the AUDIT C, the T-ACE and the CAGE screening tools for alcohol consumption. Information on smoking established the woman’s smoking history in the six months prior to pregnancy, current smoking, numbers of cigarettes smoked on average each day, attempts to quit smoking, and approaches used to quit smoking. Similar questions were repeated in the third trimester questionnaire with additional questions addressing smoking behaviour in the interval between the booking interview and the third trimester. The questions were designed to enable accurate documentation of smoking prior to pregnancy and during pregnancy.

Prior to sending the third trimester questionnaire, it was important to check the pregnancy status of all women who had been recruited to the study. At this stage 71 participants were removed from the cohort as they had become ineligible for a variety of reasons including miscarriage, molar pregnancy, multiple pregnancy, or a stated preference not to receive the third trimester questionnaire. Women who had an intrauterine death prior to the third trimester remained in the cohort in terms of recording delivery outcomes but did not receive correspondence. The remaining 1,220 eligible women were posted a third trimester questionnaire and pre-paid return addressed envelope.

One week after sending out the questionnaire, a reminder phone call was made to women who had not yet returned the questionnaire. On request from the women a further questionnaire and stamped addressed envelope was sent out. This was followed one week later by a subsequent reminder call. Follow-up phone calls were made to approximately 300 women. Women sometimes arranged to meet a member of the research team while attending their antenatal clinic and self-completed the questionnaire then. In total 907 questionnaires were returned with very little missing data. Women who booked for antenatal care but delivered elsewhere were not included in the final cohort.

The data from the booking interview and third trimester questionnaire were linked to the electronic delivery record and neonatal records with information on the mother and infant up until first hospital discharge. The medical records were reviewed for detailed information on obstetrical or neonatal complications. Information on the following maternal characteristics was extracted from the electronic records: maternal age, marital status, socioeconomic group, nationality, public or privately funded antenatal care, parity, planned pregnancy, gestation at booking, smoking, alcohol use, illicit drug use, and referral to a social worker. Maternal age was divided into the following bands: <20 years, 20–24 years, 25–29 years, 30–34 years, 35–39 years and >40 years. Socioeconomic groups were classified as professional/manager/employer, home duties, non-manual, manual, unemployed and non-classifiable. Nationality was recorded as either Irish or non-Irish and further sub-divided by region into Western Europe, Asia/Middle East, Eastern Europe, Africa, South America, North America, Australia & New Zealand. Gestational age at booking was divided into <12 weeks, 12–20 weeks and >20 weeks. Smokers were defined as women who were current smokers at the time of attendance at their first antenatal visit. Illicit drug users were defined as women who had ever used illicit drugs.

### 2.4. Antenatal Care

Each woman had a detailed booking interview with a midwife in private at the first antenatal visit. Any woman who reported smoking was given advice on smoking cessation including referral to smoking cessation support resources, including on-line resources and telephone help-lines. Nicotine replacement therapy was not routinely recommended unless prescribed by the patient’s doctor. Further advice in the second and third trimesters was at the discretion of the health professionals providing ongoing care. Every woman had an ultrasound scan at the first antenatal visit and a further detailed structural anatomy scan at 20–22 weeks gestation. Gestational age was estimated from the calculation based on first day of the last menstrual period but the booking ultrasound scan estimate was preferred if the dates were uncertain or there was a discrepancy of more than seven days.

### 2.5. Perinatal Outcomes

Perinatal outcome measures included gestational age at delivery, live birth or stillbirth, birth weight, infant gender, infant’s condition at birth including Apgar scores at 1 and 5 min, admission to the neonatal unit, any suspected congenital abnormalities and whether resuscitation was required. Detailed data on the neonate were extracted on infants admitted to the neonatal unit. Preterm birth was defined as the birth of a live baby at less than 37 weeks gestation and low birth weight was defined as weighing less than 2,500 g. Intrauterine growth restriction (IUGR) was defined as a birth weight less than the 10th percentile using individualised birth rate ratios (corrected for maternal height and weight, parity, infant sex, ethnicity and gestation) [10].

**Figure 1:**
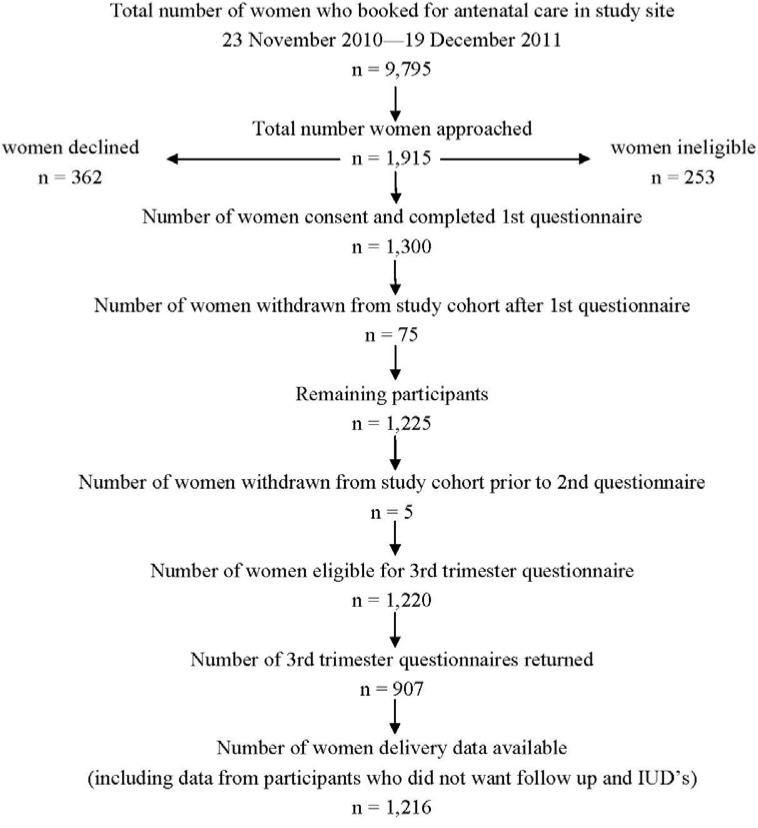
Cohort flow chart.

### 2.6. Analysis

In total 1,915 women were approached of whom 1,300 agreed to participate in the study and completed the research interview at the first hospital visit. (Figure 1) Of these 1,216 delivered in the hospital and 907 (75%) completed the third trimester questionnaire. The analyses in relation to smoking were limited to the 907 mother-infant pairs on whom data was available from pre-pregnancy through to delivery. The analyses were performed using the Statistical Package for Social Sciences (SPSS version 16). Descriptive statistics were used to characterise the study subjects by category of smoking. Comparisons were made between the three groups to identify socio-demographic factors associated with smoking cessation or continuing to smoke in pregnancy. Logistic regression analyses were performed to measure the association between smoking and adverse perinatal outcomes. The “non-smoker” category was chosen as the comparator for each of the analyses as this was unlikely to be biased by under-reporting. Further stepwise logistic regression analysis was performed adjusting for potential confounding factors including maternal age, nationality, private health insurance, unplanned pregnancy, alcohol use, and illicit drug use. These factors were chosen because of their known or possible association with adverse perinatal outcome and because of baseline differences between the groups. Results are reported as proportions, crude odds ratios (OR) and adjusted odds ratios (OR) with 95% confidence intervals (CI). All of the chosen variables for the logistic regression models are required data items on the computer system, therefore we had very little missing data, and the value “unknown” was rarely used. The study received the approval of the Coombe Women and Infants University Hospital’s research ethics committee: Study No. 22-2009.

## 3. Results

### 3.1. Descriptive Statistics

The socio-demographic characteristics of the recruited cohort, the delivery cohort and the third trimester cohort were very similar (Table 1). The study cohort was comparable to the general hospital population [9] except for the higher proportion of non-Irish participants and lower proportion of private patients reflecting higher rates of recruitment in the public clinics.

**Table 1:** Characteristics of Study Cohort in relation to the hospital population.

	Study population at recruitment ^i^	Study population at third trimester ^ii^	Study population at delivery ^iii^	General hospital population ^iv^
	n = 1,300 (%)	n = 907 (%)	n = 1,216 (%)	n = 6,720 (%)
*Maternal age at booking*				
<20 years	34 (2.6)	19 (2.1)	31 (2.5)	200 (3.0)
20–24 years	161 (12.4)	102 (11.2)	152 (12.5)	776 (11.6)
25–29 years	362 (27.8)	235 (25.9)	336 (27.6)	1,527 (22.7)
30–34 years	453 (34.8)	334 (36.8)	427 (35.1)	2,322 (34.6)
35–39 years	247 (19.0)	188 (20.7)	232 (19.1)	1,592 (23.7)
>40 years	43 (3.3)	29 (3.2)	38 (3.1)	301 (4.5)
*Marital status*				
Married	679 (52.2)	505 (55.7)	635 (52.2)	3,952 (58.5)
Single	621 (47.8)	402 (44.3)	581 (47.8)	2,685 (40.0)
*Socioeconomic group*				
Professional	341 (26.2)	258 (28.4)	317 (26.1)	2,077 (30.9)
Home duties	222 (17.1)	135 (14.9)	206 (16.9)	961 (14.3)
Non-manual	491 (37.8)	369 (40.7)	481 (39.6)	2,622 (39.0)
Manual	65 (5.0)	44 (4.9)	46 (3.8)	267 (4.0)
Unemployed	117 (9.0)	50 (5.5)	103 (8.5)	501 (7.5)
Non-classifiable	64 (4.9)	51 (5.6)	63 (5.2)	289 (4.3)
*Nationality*				
Irish	888 (68.3)	618 (68.1)	839 (69.0)	5,510 (82.0)
Non-Irish	412 (31.7)	289 (31.9)	377 (31.0)	1,189 (17.7)
Gestation at booking *				
<12 weeks	528 (40.8)	369 (40.7)	493 (40.5)	2,666 (39.8)
12–20 weeks	729 (56.3)	523 (57.7)	687 (56.5)	3,683 (55.0)
>20 weeks	37 (2.9)	15 (1.7)	36 (3.0)	349 (5.2)
*Private Health Care*				
Yes	145 (11.2)	122 (13.5)	142 (11.7)	1,219 (18.1)
No	1,155 (88.8)	785 (86.5)	1,074 (88.3)	5,499 (81.9)

^i^ Recruitment took place at participants first antenatal visit to the hospital usually at 10–14 weeks’ gestation; ^ii^ The third trimester questionnaire was completed by participants from 28 weeks’ gestation; ^iii^ Study population at delivery includes intrauterine death n = 7 and neonatal death n = 1; ^iv^ General hospital population—Murphy *et al.* (2013) [9]; * Missing data for gestational age at booking n = 6.

In the six months prior to pregnancy 270 (30%) women reported smoking and at the booking interview 160 (59%) of these had ceased smoking and 110 (41%) continued to smoke. Of the smokers almost 20% smoked more than ten cigarettes a day with very little change in smoking behaviour between the booking interview and the third trimester of pregnancy (Table 2) Of the ex-smokers 13 (8.1%) reported “any” smoking since the booking interview but all had ceased smoking by the third trimester. The current smokers reported greater use of nicotine replacement therapy or alternative therapies in their attempts to quit smoking and of the two women who had tried to quit later in pregnancy both had relapsed by the third trimester.

**Table 2:** Smoking behaviour in pregnancy.

	Ex-smoker	Current smoker
	n = 160 (%)	n = 110 (%)
*Cigarettes smoked at booking interview*	n/a	
1–5 per day		49 (44.5)
6–10 per day		41 (37.3)
11–20 per day		17 (15.5)
>20 per day		2 (1.9)
*Ever tried to quit*	148 (92.5)	83 (75.5)
Methods used to quit *		
Nicotine replacement	5 (3.1)	25 (22.7)
Alternative therapies	3 (1.9)	12 (11.1)
GP support	1 (0.6)	0 (0.0)
Self-motivated	139 (86.9)	55 (50.0)
*Any smoking since booking interview*	13 (8.1)	110 (100.0)
*Cigarettes smoked at third trimester*	n/a	
1–5 per day		45 (40.9)
6–10 per day		43 (39.1)
11–20 per day		18 (16.4)
>20 per day		3 (2.7)

* some women used more than one method.

The characteristics of the women in the cohort in relation to smoking in pregnancy are presented in Table 3. Compared to non-smokers, ex-smokers were less likely to be older OR 0.62 (95% CI 0.44, 0.89) for age 30–39 years, and more likely to be single OR 2.78 (95% CI 1.95, 3.97) or to have a history of illicit drug use OR 3.58 (95% CI 2.25, 5.69). Current smoking was associated with younger maternal age OR 0.50 (95% CI 0.33, 0.76) for age 30–39 years, less favourable socio-economic status, Irish Nationality OR 3.23 (95% CI 1.86, 5.62), unplanned pregnancy OR 1.90 (95% CI 1.26, 2.88), alcohol use in the first trimester OR 3.38 (95% CI 2.05, 5.57) and a history of illicit drug use OR 3.56 (95% CI 2.10, 6.01).

**Table 3:** Characteristics of women according to smoking in pregnancy.

Total	Non-smoker	Ex-smoker	Current smoker	Odds ratio ^i^ 95% Confidence Intervals	Odds ratio ^ii^ 95% Confidence Intervals
n = 907	n = 637 (%)	n = 160 (%)	n = 110 (%)		
*Maternal age*					
<20 years	13 (2.0)	3 (1.9)	3 (2.7)	0.68 (0.19, 2.44)	0.90 (0.25, 3.28)
20–29 years ^∫^	211 (33.1)	72 (45.0)	54 (49.1)	1.00	1.00
30–39 years	389 (61.1)	83 (51.9)	50 (45.5)	0.62 (0.44, 0.89) *	0.50 (0.33, 0.76) *
>40 years	24 (3.8)	2 (1.3)	3 (2.7)	0.24 (0.06, 1.06)	0.50 (0.14, 1.68)
*Single Marital status*	223 (35.0)	96 (60.0)	83 (75.5)	2.78 (1.95, 3.97) *	5.71 (3.59, 9.07) *
*Socioeconomic* *group*					
Professional	211 (33.1)	39 (24.4)	8 (7.3)	0.66 (0.37, 1.17)	0.10 (0.05, 0.24) *
Home duties ^∫^	82 (12.9)	23 (14.4)	30 (27.3)	1.00	1.00
Non-manual	250 (39.2)	75 (46.9)	44 (40.0)	1.07 (0.63, 1.82)	0.48 (0.28, 0.82) *
Manual	33 (5.2)	5 (3.1)	6 (5.5)	0.54 (0.19, 1.54)	0.50 (0.19, 1.30)
Unemployed	23 (3.6)	11 (6.9)	16 (14.5)	1.71 (0.73, 4.01)	1.90 (0.89, 4.08)
Non-classifiable	38 (6.0)	7 (4.4)	6 (5.5)	0.66 (0.26, 1.66)	0.43, (0.17, 1.12)
*Irish Nationality*	411 (64.5)	113 (70.6)	94 (85.5)	1.32 (0.91, 1.93)	3.23 (1.86, 5.62) *
*Private Health Care*	103 (16.2)	16 (10.0)	3 (2.7)	0.58 (0.33, 1.01)	0.15 (0.05, 0.47) *
*Nulliparous*	282 (44.3)	69 (43.1)	46 (41.8)	0.96 (0.67, 1.35)	0.91 (0.60, 1.36)
*Unplanned pregnancy*	189 (29.7)	50 (31.3)	49 (44.5)	1.08 (0.74, 1.57)	1.90 (1.26, 2.88) *
*Gestation at booking*					
<12 weeks ^∫^	255 (40.0)	66 (41.3)	48 (43.6)	1.00	1.00
12–20 weeks	374 (58.7)	89 (55.6)	60 (54.5)	0.92 (0.64, 1.31)	0.85 (0.57, 1.29)
>20 weeks	8 (1.3)	5 (3.1)	2 (1.8)	2.41 (0.77, 7.62)	1.33 (0.27, 6.45)
*Alcohol first trimester*	61 (9.6)	15 (9.4)	29 (26.4)	0.98 (0.54, 1.77)	3.38 (2.05, 5.57) *
*Illicit drug use (ever)*	51 (8.0)	38 (23.8)	26 (23.6)	3.58 (2.25, 5.69) *	3.56 (2.10, 6.01) *
*Social worker referral*	12 (1.9)	4 (2.5)	6 (5.5)	1.35 (0.43, 4.25)	3.00 (1.10, 8.18) *

^i^ Ex-smoker *versus* Non-smoker; ^ii^ Current smoker *versus* Non-smoker; ^∫^ Reference category; * *p* < 0.05.

### 3.2. Perinatal Outcomes

Ex-smokers had very similar perinatal outcomes to non-smokers with no significant difference in average birth weight or incidence of intrauterine growth restriction (Table 4). Compared to non-smokers, current smoking was associated with an average reduction in birth weight of 191 g (95% CI −294, −88) and an increased incidence of intrauterine growth restriction (IUGR) (21.8% *versus* 14.9%, crude OR 2.09 (95% CI 1.27, 3.44). The association was attenuated on controlling for potential confounding factors, adjusted OR 1.39 (1.06, 1.84). Current smokers who smoked more than 10 cigarettes a day had babies with an average birth weight of 237 g less (95% CI −485, 10) than women smoking 1–5 cigarettes a day.

**Table 4:** Perinatal outcomes according to reported smoking behaviour at booking visit and third trimester of pregnancy.

Alcohol intake	Non-smoker n = 637	Ex-smoker n = 160	Current smoker n = 110	Ex-smoker *versus* Non-smoker Mean difference (95% CI) Crude OR (95% CI) Adjusted ^ii^ OR (95% CI)	Current smoker *versus* Non-smoker Mean difference (95% CI) Crude OR (95% CI) Adjusted ^ii^ OR (95% CI)
Gestational age (weeks) Mean (SD) Range	39.7 (1.5) 29–42	39.9 (1.5) 32–42	39.6 (1.4) 36–41	0.2 (−0.1, 0.5)	−0.1 (−0.4, 0.2)
Birth weight (g) Mean (SD) Range	3,496 (509) 1,145–5,160	3,503 (491) 1,870–4,805	3,305 (491) 2,120–4,700	7 (−81, 95)	−191 (−294, −88) *
Preterm birth < 37 weeks (%)	28 (4.4)	8 (5.0)	6 (5.5)	1.14 (0.51, 2.56) 1.68 (0.51, 5.630	1.25 (0.51, 3.10) 1.09 (0.68, 1.75)
Low birth weight < 2,500 g (%)	21 (3.3)	5 (3.1)	5 (4.5)	0.95 (0.35, 2.55) 1.09 (0.37, 3.21)	1.28 (0.47, 3.45) 1.24 (0.73, 2.09)
Intrauterine growth restriction ^1^ (%)	82 (12.9)	17 (10.6)	26 (23.6)	0.81 (0.46, 1.40) 1.05 (0.58, 1.89)	2.09 (1.27, 3.44) * 1.39 (1.06, 1.84) *
Apgar score < 7 at 5 min (%)	3 (0.5)	3 (1.9)	1 (0.9)	4.04 (0.81, 20.2) 4.30 (0.81, 22.8)	1.94 (0.20, 18.8) 1.22 (0.38, 3.94)
Admitted to neonatal unit (%)	95 (14.9)	29 (18.1)	24 (21.8)	1.26 (0.80, 2.00) 1.34 (0.76, 2.14)	1.59 (0.96, 2.63) 1.66 (0.94, 2.83)

^1^ Customised birth weight < 10th percentile; ^ii^ Adjusted for maternal age, Body Mass Index (BMI, except for IUGR), Irish nationality, unplanned pregnancy, private healthcare, alcohol use, illicit drug use; * *p* < 0.05.

## 4. Discussion

### 4.1. Summary of Main Findings

This study found that almost 60% of prior smokers attending for antenatal care had made a decision to quit smoking by the time of the first antenatal visit and that ex-smokers have perinatal outcomes similar to non-smokers. Most women who quit smoking were self-motivated to do so and there was very little change in smoking behaviour between the first and third trimesters of pregnancy. Continued smoking in pregnancy was associated with social disadvantage, alcohol consumption in pregnancy and a history of illicit drug use. Smoking in pregnancy was associated with an average birth weight reduction of 191g and a doubling in the incidence of intrauterine growth restriction.

### 4.2. Strengths and Limitations of the Study

The study population consisted of a representative cohort of women attending a large urban maternity hospital between 2010 and 2011. The data were collected prospectively by qualified health researchers using a standardised interview schedule at the first visit and a self-completed questionnaire in the third trimester. This was supplemented by a computer guided interview completed by the midwife conducting the booking history. Therefore, we had detailed information on lifestyle behaviours ascertained three different ways at two separate time points. As the data were collected at the first antenatal visit and again in the third trimester of pregnancy the potential for recall bias was limited. Nonetheless the data on smoking relied on self-reporting by the pregnant woman and it is possible that cigarette smoking was under-reported. Despite written reminders and telephone contact there was a loss of responders in the third trimester, however the profile of the cohort at the first interview and in the third trimester suggests that the loss to follow-up was random rather than specific to a particular sub-group of patients. Although more costly, it emphasises the importance of direct patient contact when conducting research on sensitive exposures in pregnancy. It was not feasible to approach all women booking for antenatal care at the hospital and some of the women approached declined to participate. It always possible that the behaviours and outcomes of those who do not participate in research may differ from those who do, nonetheless we were satisfied that we sampled a broad spectrum of pregnant women.

### 4.3. Comparison with Existing Literature

Similar to other studies in Europe, the United States of America, Australia and New Zealand, we found that nearly one in eight women continues to smoke in pregnancy [3,6,11,12]. A smoking cessation rate of 60% at the first antenatal visit compares favourably to 40% cessation rates among women in the United States [5,13,14]. We have confirmed the known associations between smoking in pregnancy and unfavourable socio-economic factors, alcohol consumption and history of illicit drug use [5,6,9,15]. A linked cohort study from Australia and New Zealand (SCOPE) reported that for healthy women having their first baby, quitting smoking before 15 weeks’ gestation significantly reduced rates of spontaneous preterm birth and small for gestational age compared to non-smokers [12]. An accompanying editorial called for these findings to be verified observationally using data from other birth cohorts [16]. Our study, in an unselected population of nulliparous and parous women, confirms the findings in relation to small for gestational age but not for preterm birth. Given the low numbers in our study affected by preterm birth it is possible that we were under-powered to establish or exclude an association with preterm birth within this population.

Understanding women’s attitudes and behaviour is essential in order to design and implement effective health promotion strategies for pregnancy. Similar to our study, others have found a marked change in healthy lifestyle behaviours in response to pregnancy and this appears to be largely self-motivated [17]. Smoking cessation is likely to be more challenging among those who have failed to self-motivate by the first antenatal visit. Although there is good evidence from a systematic review that interventions in pregnancy can reduce maternal smoking, cluster randomised trials of midwife led interventions have not been shown to be effective [5,18]. This may reflect reservations midwives have about introducing smoking cessation at the first antenatal visit when they are trying to establish a relationship with women, or the dilutional effect of addressing a wide range of healthcare issues at a single consultation. However, given the success reported by women in continuing to abstain from smoking having quit in the first trimester, it is essential that further efforts are made to encourage smoking cessation at all stages of pregnancy.

### 4.4. Implications for Practice

Public Health campaigns emphasise the health benefits of smoking cessation in pregnancy [19,20]. The greatest success within our cohort was pre-pregnancy and during the first trimester, where women were largely self-motivated to quit. Further attention needs to be focussed on women who continue to smoke throughout pregnancy and on interventions that support them to quit successfully. In particular, smoking cessation strategies need to take account of the competing health promotion initiatives that are part of routine antenatal care, such as folic acid supplementation, eating a balanced diet, taking regular exercise, achieving appropriate weight gain, preparing for childbirth and breast feeding. Although the greatest perinatal advantage appears to relate to early sustained smoking cessation in pregnancy, later cessation will have advantages for the infant environment and could be carried forward to subsequent pregnancies. 

## 5. Conclusions

Almost 60% of prior smokers quit either pre-pregnancy or in the first trimester of pregnancy and most are self-motivated to do so. These women have perinatal outcomes similar to non-smokers. Continued smoking in pregnancy is associated with lower birth weight and intrauterine growth restriction. Women who have failed to quit smoking are more likely to have tried nicotine replacement therapy or alternative therapies. The ongoing challenge for health professionals providing antenatal care is to find effective strategies to support smoking cessation among the 10% to 12% of women who find it difficult to quit, many of whom have less advantageous personal circumstances.
